# High brightness formamidinium lead bromide perovskite nanocrystal light emitting devices

**DOI:** 10.1038/srep36733

**Published:** 2016-11-09

**Authors:** Ajay Perumal, Sushant Shendre, Mingjie Li, Yong Kang Eugene Tay, Vijay Kumar Sharma, Shi Chen, Zhanhua Wei, Qing Liu, Yuan Gao, Pio John S. Buenconsejo, Swee Tiam Tan, Chee Lip Gan, Qihua Xiong, Tze Chien Sum, Hilmi Volkan Demir

**Affiliations:** 1 LUMINOUS! Center of Excellence for Semiconductor Lighting and Displays, School of Electrical and Electronic Engineering, Nanyang Technological University, 639798, Singapore; 2 Division of Physics and Applied Physics, School of Physical and Mathematical Sciences, Nanyang Technological University, 637371, Singapore; 3 UNAM-Institute of Materials Science and Nanotechnology, Department of Electrical and Electronics Engineering, Department of Physics, Bilkent University, Ankara, 06800, Turkey; 4 School of Materials Science and Engineering, Nanyang Technological University, 639798, Singapore

## Abstract

Formamidinium lead halide (FAPbX_3_) has attracted greater attention and is more prominent recently in photovoltaic devices due to its broad absorption and higher thermal stability in comparison to more popular methylammonium lead halide MAPbX_3_. Herein, a simple and highly reproducible room temperature synthesis of device grade high quality formamidinium lead bromide CH(NH_2_)_2_PbBr_3_ (FAPbBr_3_) colloidal nanocrystals (NC) having high photoluminescence quantum efficiency (PLQE) of 55–65% is reported. In addition, we demonstrate high brightness perovskite light emitting device (Pe-LED) with these FAPbBr_3_ perovskite NC thin film using 2,2′,2″-(1,3,5-Benzinetriyl)-tris(1-phenyl-1-H-benzimidazole) commonly known as TPBi and 4,6-Bis(3,5-di(pyridin-3-yl)phenyl)-2-methylpyrimidine (B3PYMPM) as electron transport layers (ETL). The Pe-LED device with B3PYMPM as ETL has bright electroluminescence of up to 2714 cd/m^2^, while the Pe-LED device with TPBi as ETL has higher peak luminous efficiency of 6.4 cd/A and peak luminous power efficiency of 5.7 lm/W. To our knowledge this is the first report on high brightness light emitting device based on CH(NH_2_)_2_PbBr_3_ widely known as FAPbBr_3_ nanocrystals in literature.

Lead halide based perovskite materials have remarkable optoelectronic properties like high absorption coefficient, large mobility and easier bandgap tunability^1^,^2^,^3^,^4^,^5^,^6^,^7^,^8^. There is an exponential surge in interest, in these class of materials especially in photovoltaic devices^6^,^9^,^10^,^11^,^12^,^13^. The semiconducting lead halide based perovskite system in particular the organic-inorganic hybrid system, namely methyl ammonium lead halide CH_3_NH_3_PbBr_3_ (MAPbX_3_)^11^,^12^,^13^ has shown great promise and photovoltaic devices with power conversion efficiency in excess of 20% have been reported^14^,^15^. Recently, formamidinium lead halide CH(NH_2_)_2_PbX_3_ (FAPbX_3_) perovskite^16^,^17^ has gained prominence with higher thermal stability in comparison to more popular methylammonium lead halide perovskite and photovoltaic devices with power conversion efficiencies in excess of 20% have been reported with FAPbI_3_^15^.

Besides their application in photovoltaic devices the organic-inorganic lead halide based hybrid perovskites have shown excellent optoelectronic properties for their application in passive photo luminescent or active electroluminescent optoelectronic devices due to their large absorption coefficients, almost no defects, and reduced Auger recombination^6^,^18^,^19^,^20^,^21^,^22^,^23^,^24^,^25^,^26^,^27^,^28^,^29^,^30^,^31^. High quality polycrystalline films can be easily processed at room temperature in ambient via simple solution based techniques and does not require any high temperature processing steps^6^,^7^,^8^,^32^. The emission wavelength of the hybrid perovskite material can be tuned over the entire visible to infrared region of the electromagnetic spectrum by simple halide cation exchange or by inter mixing of halide cations, making them an excellent class of materials especially for their use in low-cost and large area optoelectronic device related applications^3^,^21^,^33^. As early as 1990s, the long-chain layered 2 dimensional alkyl organic/inorganic lead halide perovskite (C_6_H_5_C_2_H_4_NH_3_)_2_PbI_4_^34^,^35^ was used in LED devices operating at liquid-nitrogen temperatures. Later, perovskite LED device was also demonstrated with (H_3_NC_2_H_4_C_16_H_8_S_4_C_2_H_4_NH_3_)PbX_4_ (X=Cl, Br, I) which operates at room temperature with power efficiency of 0.1 lm/W^36^. Friend’s group reported infrared emitting LED devices with low turn on voltage (1.5 V) and high external quantum efficiency (EQE) of 0.76% by using short chain perovskite material MAPbX_3_ as an active layer^25^. More recently, Tae Woo Lee and co-workers have demonstrated thin film perovskite green emitting LED device with EQE of 8.5% and power efficiency of 42.9 cd/A with MAPbBr_3_ thin films, demonstrating the potential of perovskites in high efficiency LED devices^37^. Their potential application in lasing^38^,^39^,^40^ and light emitting transistor (LET)^41^ have also been reported.

Recently, a lot of progress has been made in the synthesis of perovskite nanocrystals (NCs) adapting similar synthesis procedures to that of inorganic semiconductor nanocrystals. Perovskite NCs exhibiting high photoluminescence quantum efficiency (PLQE) and high color purity have been reported^22^,^42^,^43^,^44^. The quantum confinement effects, in perovskite nanostructures are not as prominent as in inorganic semiconductor nanocrystals^43^,^45^,^46^. The NC size variation does not result in tuning of emission wavelength over entire visible range of the electromagnetic spectrum. The color tuning is much more pronounced and controlled in perovskite NCs via cation replacement or intermixing of the cation with different halides^3^,^33^,^43^,^44^. Previously, methyl ammonium lead halide and cesium lead halide based nanocrystals have been employed for fabricating light-emitting devices^19^,^20^,^24^,^47^.

Herein, we report a simple, easy to perform and highly reproducible room temperature synthesis of device grade, polycrystalline and highly luminescent FAPbBr_3_ colloidal NC having a high PLQE of 55–65%. These perovskite NC’s exhibit bright photoluminescence (PL) with an emission peak centred at 531.3 nm having a very narrow full width at half maximum (FWHM) of 21.6 nm. The PLQE value reported is 10 fold improvement in comparison to prior published literature for FAPbBr_3_ NP synthesis (PLQE of 5%), using them light emitting electrochemical cells were demonstrated with maximum light output of 1–2 cd/m^2 ^48. We demonstrate high brightness LEDs based on solution cast FAPbBr_3_ nanocrystals, and the Pe-LED devices have bright electroluminescence of up to 2714 cd/m^2^, luminous efficiency of 6.4 cd/A and luminous power efficiency of 5.7 lm/W. This is the first report on high brightness light emitting diode device based on FAPbBr_3_ nanocrystals and to our knowledge, till date there is no report on efficient Pe-LEDs using FAPbBr_3_ NCs in literature. Also with temperature dependent exciton dynamics, we propose exciton quenching in perovskite nanocrystals especially the roll off in luminance and efficiency at higher voltages which limits the performance of perovskite LED devices.

## Results and Discussion

**Figure 1:**
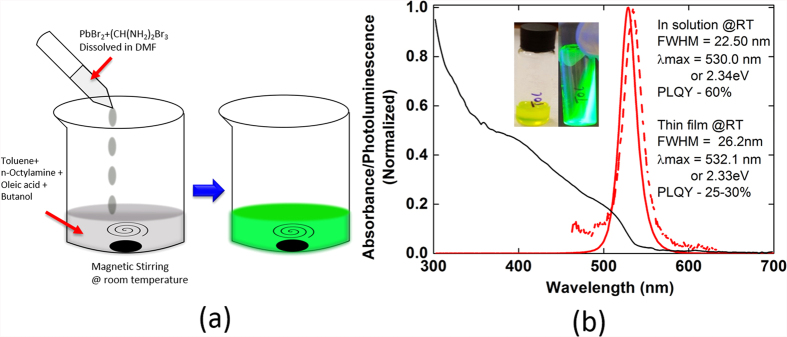
(**a**) Brief schematic illustration of the synthesis procedure where the precursor consisting of PbBr_2_ and CH(NH_2_)_2_Br dissolved in DMF solvent is added dropwise to toluene consisting of ligands and stabilizer under vigorous stirring at room temperature. (**b**) The absorption and PL spectra of FAPbBr_3_ nanocrystals in toluene solution, for reference the thin film PL is also overlaid on the solution PL. The inset shows FAPbBr_3_ NC in toluene under ambient light and under UV exposure.

The synthesis procedure for FAPbBr_3_ NCs is similar to that of MAPbBr_3_ nanocrystals reported earlier by Feng Zhang and co-workers^22^. Briefly, a precursor solution consisting of CH(NH_2_)_2_Br and PbBr_2_ dissolved in N-dimethylformamide (DMF) is added dropwise into a solution consisting of toluene and organic long chain ligands (n-Octylamine) and a stabilizer (oleic acid) under constant stirring. The transparent toluene solution with ligands and stabilizer turns to greenish yellow in color after the precursor addition, confirming the formation of colloidal perovskite nanocrystals as shown in Fig. 1(a).

The greenish yellow colloidal perovskite nanocrystal solution is centrifuged at 7840 rpm for 5 minutes and the supernatant is discarded. The bottom precipitate is again re-dissolved in fresh anhydrous toluene and the solution is centrifuged again for 5 minutes at 7840 rpm, this time the supernatant is collected for device fabrication and the bottom precipitate is discarded. Figure 1(b) shows the absorption (black) and emission (red) spectra of FAPbBr_3_ NCs dispersed in toluene. The PL emission in toluene is roughly centred on the onset of absorption peak with PL peaked at 530.0 nm (2.34 eV) having a narrow full width half maximum (FWHM) of 22.5 nm. The thin film PL emission at room temperature has PL emission peak at 532 nm which is slightly red shifted in comparison to solution PL emission peak and has FWHM of 26 nm slightly broader compared to solution FWHM. The PL emission is mainly due to exciton recombination in these materials as there is very little Stokes shift between absorption peak (first exciton peak) and the PL emission peak in perovskite NCs. The bulk FAPbBr_3_ films have been reported to have PL emission peak >550 nm^16^,^17^,^49^,^50^, hence the emission peak for FAPbBr_3_ NCs is blue-shifted by 10–20 nm compared to that of FAPbBr_3_ bulk materials. The FAPbBr_3_ NC solution in ambient light and under UV-excitation is shown in the inset of Fig. 1(b). Strong PL is observed from FAPbBr_3_ NCs under UV light for both NCs in solution and in thin film form. The synthesis procedure is highly reproducible with PLQE in solution between 55–65% for different batch synthesis of perovskites as shown in Supplementary info Figure S6.

**Figure 2:**
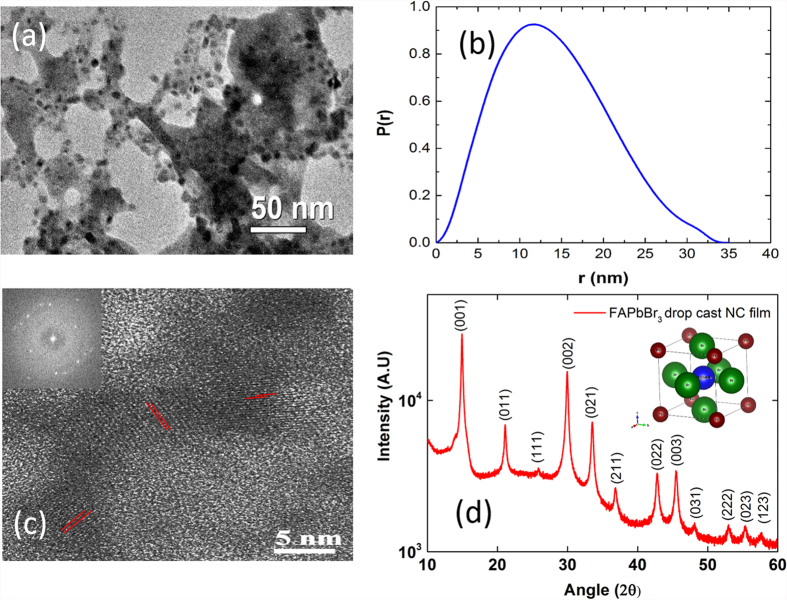
(**a**) Transmission electron microscopy (TEM) image of FAPbBr_3_ NCs. The black dots correspond to FAPbBr_3_ NCs having an average size of 10–15 nm. (**b**) Particle size distributions measured via small angle x-ray scattering technique (SAXS) the as synthesized FAPbBr_3_ NCs with an average size of 12–14 nm. (**c**) The high-resolution transmission electron microscopy (HR-TEM) of the FAPbBr_3_ NCs with its fast Fourier transformation (FFT) image shown in the inset. (**d**) X-ray diffraction (XRD) patterns for drop cast FAPbBr_3_ NCs.

The as-prepared perovskite NCs are primarily polycrystalline having broad size distribution. Figure 2(a) shows the transmission electron microscopy (TEM) image of FAPbBr_3_ NCs. The FAPbBr_3_ NCs have an average size of 10–15 nm. We have also measured the particle size distribution via small angle x-ray scattering technique and the as synthesized FAPbBr_3_ NCs have particle distribution from 1–30 nm with an average size of 12–14 nm shown in Fig. 2(b). The average size obtained from SAXS are consistent with the TEM measurements. Further information as to how to formulate the particle size from SAXS is described in Supplementary Information. We can also resolve the crystalline lattice planes of these FAPbBr_3_ NCs in the high resolution transmission electron microscopy (HR-TEM) image shown in (Fig. 2(c)) with its fast Fourier transformation (FFT) image shown in the inset of Fig. 2(c). The TEM measurements confirm high degree of crystallinity in these FAPbBr_3_ NCs. X-ray diffraction (XRD) pattern for drop cast FAPbBr_3_ NCs is recorded at room temperature and the XRD spectra is shown in Fig. 2(d).

The XRD data clearly shows that the NCs are highly polycrystalline. The XRD patterns are indexed to the cubic phase of FAPbBr_3_ with a unit cell of 6.006 Å with Pm-3 m space group, similar to that of bulk FAPbBr_3_ perovskite^17^,^51^. The detailed indexing is shown in Table ST1 of supplementary info. The inter-planar distance of ~3 Å from the HR-TEM image correspond to the 002 plane of crystal orientation in XRD analysis. According to temperature dependent PL studies performed for MAPbBr_3_ bulk films, it is reported that at room temperature the MAPbBr_3_ bulk film is in tetragonal phase^52^, there is phase transition from tetragonal to cubic phase at 320 K. Due to similarities in MAPbBr_3_ and FAPbBr_3_ structures, we propose that the FAPbBr_3_ to be in tetragonal phase at room temperature. However, the XRD measurements and indexing performed at room temperature indicate the FAPbBr_3_ NC film to be in cubic phase as the XRD peaks are indexed to cubic phase (tetragonal indexing for XRD measurements resulted in lattice constants a and b to be very similar). From temperature dependent PL studies performed on MAPbBr_3_ and assuming the phase transitions for MAPbBr_3_ and FAPbBr_3_ to be similar, we believe that it is difficult to differentiate between these two phases.

Temperature dependent exciton dynamics studies are performed to extract exciton binding energy and to understand PL dynamics as a function of temperature for FAPbBr_3_ NC thin films. The exciton binding energy is directly related to the absorption and emission of an optoelectronic material.

**Figure 3:**
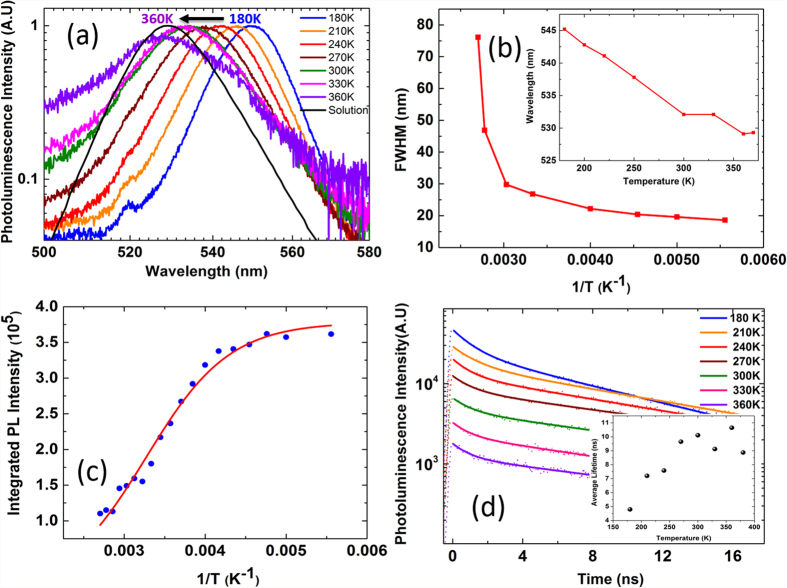
(**a**) The normalized PL emission spectra under a pump fluence of 1.2 μJ cm^−2^ at temperatures ranging from 180–360 K. The PL spectra are normalized at their maximum intensity. (**b**) The change in FWHM is shown as a function of inverse temperature and in the inset the shift in the PL emission peak is shown as a function of temperature. (**c**) The integrated PL intensity as a function of inverse temperature and the Arrhenius fit (solid line) for temperature range 180–360 K (**d**) The TRPL intensity and the fit along with the average lifetime (inset) is shown for temperature range of 180–360 K.

Figure 3(a) shows normalized PL spectra for the temperature range from 180–360 K for FAPbBr_3_ NC thin film under an excitation pump fluence of 1.2 μJ cm^−2^. We restrict our temperature dependent studies to 180 K as the low temperature limit due to experimental constraints and we believe below 180 K the low temperature PL exciton dynamics to be similar to the MAPbBr_3_ thin films reported earlier^52^. The FWHM increases as the temperature increases as shown in Fig. 3(b) and this is attributed to increased phonon interactions and phonon scattering^53^,^54^. The thin film PL at 180 K is narrower with FWHM of 18.6 nm, while at 360 K the FWHM of the PL is 46.9 nm. There is red shift in PL peak position with lowering temperature consistent with FAPbBr_3_ bulk thin films and other lead halide perovskites^49^,^53^,^54^, the PL peak is at ~530 nm at room temperature and the PL peak shifts to ~545 nm at 180 K as shown in the inset of Fig. 3(b). This is attributed to lattice strain^53^, contraction of the lattice and reorientation of crystallites in the lattice^54^. The integrated PL emission intensity (area under the PL peak at different temperatures) versus the inverse of temperature for FAPbBr_3_ NCs and the Arrhenius fits in the temperature range 180–360 K is shown in Fig. 3(c).

We also observe significant reduction in the absolute PL intensity with increase in temperature as shown in Figure S1 of supplementary info. The nonradioactive recombination is thermally activated in FAPbBr_3_ and is increasing with the temperature. We can extract exciton binding energy of these nanocrystals by fitting the curve with following equation^55^,
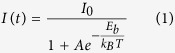
where I_0_ is the emission intensity extrapolated at 0 K, E_b_ is the exciton binding energy, and k_B_ is the Boltzmann constant.

We find that the exciton binding energy of FAPbBr_3_ NCs we synthesized is roughly 170 meV (Fig. 3(c)) which is much higher than the reported exciton binding energies of the bulk thin films^49^. We have measured the time-resolved PL spectra for FAPbBr_3_ NCs in thin film and in solution as shown in Fig. 3(d) for temperature range 180–360 K. The PL decay transients can be fitted with the bi-exponential of the form^23^,
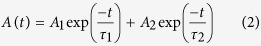
For both thin film and solution, we observe bi-exponential decay resulting in two time constants one with the shorter lifetime, which we attribute to NCs whose recombination is strongly mediated by surface impurities and traps and the longer lifetime due to intrinsic band edge emission. The relative contribution of these two terms to the PL emission in solution shows that the fast component has only 20% contribution of the total PL emission and the faster lifetime component is ~5 ns. The rest 80% contribution of the PL emission is due to band edge emission and the corresponding slower component lifetime is ~69 ns. The TRPL for NCs in solution is shown in supplementary info Figure S3. For NC thin films, the faster component proportion roughly doubles in comparison to the solution sample from 20% to 32% at room temperature. The surface of the NCs is exposed in case of thin films and the surface trap density is much higher than in the case of the solution. The shorter lifetime τ_1_ is decreasing with increasing temperatures due to possible non-radiative recombination. The slower component proportion is reduced from 80% in solution to ~70% in NC thin films at room temperature. The longer component τ_2_ increases with increasing temperature a similar to the trend that is observed in CdSe nano sheets, which is attributed to the giant oscillator strength (GOST) effect in low dimensions^56^. Although the average lifetime increases as a function of increasing temperature as shown in Table 1 and the inset of Fig. 3(d), the absolute PL signal intensity significantly decreases (shown in Figure S1 of supplementary info). This implies the PL quantum yield decreases as a function of increasing temperature, which is consistent with previous report in perovskite bulk films^57^. The average lifetime of FAPbBr_3_ NCs film is much shorter in comparison to the solution sample, at room temperature NC thin film has average lifetime of 10 ns with PLQE roughly 25%. The solution of FAPbBr_3_ in toluene on the other hand has much larger average lifetime of 55 ns and also much higher PLQE of 55–65%.

We can estimate the exciton Bohr radius for these FAPbBr_3_ NC system from the following equation,
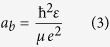
where ε is the static dielectric constant, ħ is the Planck’s constant 1.05 ∗ 10^−34^ Js, μ is the reduced exciton effective mass (the bulk value 0.13 m_0_^58^) and e is unit charge of electron 1.6 ∗ 10^−19^ Js coulomb. The perovskite NC film is not dense in comparison to bulk perovskite film and hence the static dielectric constant value will be lower in comparison to bulk. The estimated Bohr radius is dependent on the value of the static dielectric constant. The exciton is also screened by phonons and random distribution of polar organic cation hence the value of ε is not easy to estimate in these films. With ε of ~20 (for MAPbI_3_ bulk films the static ε is reported to be in the range of 35–70^59^), we estimate the Bohr exciton radius to be 8 nm much larger than the lattice constant. We do not observe the strong quantum confinement effect in these nanocrystals as a function of size due to their relatively larger size. This observation is supported by the PL emission measurements of supernatant (having nanocrystals of smaller size) and the precipitate (having nanocrystals of much larger size compared to supernatant), their emission maxima does not have strong shift as shown in supporting info Figure S2.

**Figure 4:**
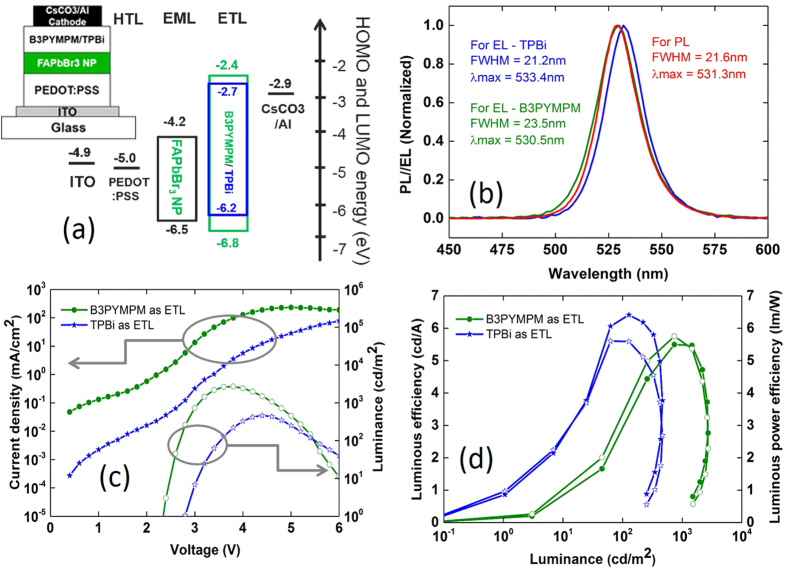
(**a**) The device structure for the Pe-LED device and the corresponding schematic energy level diagram for the materials. (**b**) EL spectra for Pe-LED device with B3PYMPM (green line) and TPBi (blue line) as ETL are shown in Fig. 4 recorded at 0.5 mA/cm^2^. The PL spectrum of FAPbBr_3_ (red line is also shown for reference). (**c**) Current density/Luminance vs Voltage (J-V-L) characteristics for Pe-LED device with B3PYMPM (green line circles) and TPBi (blue line stars). (**d**) Luminous efficiency (cd/A) vs luminance (cd/m^2^) and Luminous power efficiency (lm/W) vs luminance (cd/m^2^) characteristics for Pe-LED device with B3PYMPM (green line circles) and TPBi (blue line stars). The filled symbols correspond to luminous efficacy cd/A and open symbols correspond to lm/W.

We fabricate the Pe-LED device with these FAPbBr_3_ NCs. The device structure and the flat band energy level diagram is shown in Fig. 4(a). The Pe-LED device with FAPbBr_3_ perovskite NCs consists of 40 nm thick poly(ethylenedioxythiophene):polystyrene (PEDOT:PSS) as hole transport/injection layer, FAPbBr_3_ NC layer and ~50 nm of B3PYMPM or TPBi as an electron transport layer with CsCO_3_ (5 nm)/Al(100 nm) cathode layer. To observe any discontinuity in perovskite film and to see the thin film morphology, we perform the cross-sectional TEM imaging of the complete Pe-LED device. Figure S5 of Supplementary Information shows a cross-sectional TEM image of the Pe-LED device, with ITO and aluminum layers clearly identified. The HTL, perovskite EML and ETL in the device stack are not resolved as there is insufficient scattering contrast between them as there is dominant carbon content in all these films. The energy levels for the FAPbBr_3_ NC layer are derived from UPS measurement shown in Figure S4 (Supplementary Information) and the energy level values for indium tin oxide (ITO) acting as semi-transparent electrode, PEDOT:PSS hole injection/transport layer (HIL/HTL), B3PYMPM and TPBi electron transport layer (ETL) are taken from refs 20 and 60. The normalized EL spectra of the perovskite LED device with TPBi and B3PYMPM as ETL, the normalized PL spectrum of FAPbBr_3_ NC film is shown in Fig. 4(b). The EL emission is due to radiative recombination of excitons in FAPbBr_3_ NCs as the EL and PL spectra overlap and the small shift in the EL spectra for Pe-LED with TPBi layer is due to optical detuning of cavity from emission maxima. This is evident from the change in the EL spectra with the change in ETL.

The refractive index of B3PYMPM and TPBi are slightly different, hence for the device with the same thickness of ETL while keeping the other layers/thicknesses same results in an EL emission which is slightly red shifted. The FWHM of the EL spectrum is slightly broader in comparison to the PL spectra of neat FAPbBr_3_ thin film. The FAPbBr_3_ PL emission has a FWHM of 22.5 nm with ~λ_max_ of 530 nm. The EL emission spectra of the Pe-LED device with TPBi as ETL has a FWHM of 21.2 nm with ~λ_max_ of 533.4 nm while the Pe-LED device with B3PYMPM as ETL has a FWHM of 23.5 nm with ~λ_max_ of 530.7 nm. The current density-voltage-luminance curve for Pe-LED devices with TPBi and B3PYMPM as ETL layers is shown in Fig. 4(c). The turn-on voltages (corresponds to luminance of 1 cd m^−2^) are 2.8 and 2.3 V for Pe-LED devices with TPBi and B3PYMPM ETLs.

The luminance-voltage plot is similar for both ETLs. Initially, the luminance increases as a function of increasing voltage until 465 cd m^−2^ at 4.4 V (for TPBi ETL) and 2714 cd m^−2^ at 3.8 V (for B3PYMPM ETL) beyond the luminance maximum the increase in voltage results in decrease of luminance. The B3PYMPM ETL has superior electron mobility and better energetic alignment^61^, hence higher current densities in the Pe-LED device therefore resulting in higher luminance. The deeper HOMO of the B3PYMPM also helps in tighter exciton confinement, hence the device with B3PYMPM has a lower onset voltage. The Pe-LED device with TPBi as ETL has lower current density, lower luminance and superior efficiency values. The luminous and luminous power efficiency as a function of the luminance is shown in Fig. 4(d).

We achieve maximum luminous efficiency of 6.4 cd A^−1^ and a maximum luminous power efficiency of 5.6 lmW^−1^ for the Pe-LED device with TPBi as ETL. For the Pe-LED device with B3PYMPM as ETL, we achieve a maximum current efficiency of 5.4 cd A^−1^ and a maximum luminous efficiency of 5.7 lmW^−1^. There is a severe roll off in luminance and device efficiency beyond 3.8 V (98 mA/cm^2^) or the maximum luminance of 2714 cd/m^2^ for the device with B3PYMPM as ETL and 4.8 V (16 mA/cm^2^) or the maximum luminance of 434 cd/m^2^ for the device with TPBi as ETL. The Pe-LED device with both B3PYMPM and TPBi has a charge imbalance evident from the increase in efficiency until there is charge balance in the device. The sudden roll-off thereafter is severe and this roll off is similar in Pe-LED devices with different ETLs. The difference in the magnitude of the current density values (higher current density with B3PYMPM and lower current density with TPBi) does not have an influence on the quenching and efficiency roll off. The PLQE measured as a function of input laser power shown in Supplementary info Figure S7, for perovskite thin film has roughly same value. Implying the exciton quenching due to higher exciton density is less significant in Pe-LED devices. Hence, the charge injection seems to result in degradation of perovskite material ultimately causing the efficiency roll off. We compare typical current density, luminance and efficiency values for a quantum dot light emitting device (QD-LED) with similar device architecture except the difference in emission layer. The quantum dots have similar PLQE values ranging from 65–70%. The device performance values for both QD-LED device and the Pe-LED device are tabulated in Table 2 at 100, 1000 and 10000 cd/m^2^. The QD-LED device shows much higher luminance and efficiency values at lower current density in comparison to Pe-LED device. Hence, we believe better material engineering is essential to achieve higher efficiency in perovskite NC LED devices, our results clearly demonstrate that with the better perovskite material having lesser PL quenching at room temperature it should be possible to achieve much higher efficiency in Pe-LED devices.

## Conclusions

In conclusion, we have demonstrated highly reproducible room temperature synthesis of device grade high quality formamidinium lead bromide (FAPbBr_3_) colloidal nanocrystals (NC). The perovskite NC synthesized have high PLQE of 55–65% and have very narrow FWHM of ~20 nm suitable for display applications with high color purity. We also demonstrate high brightness perovskite light emitting device (Pe-LED) with these FAPbBr_3_ perovskite NC thin film using TPBi and B3PYMPM as electron transport layers (ETL). The Pe-LED device with TPBi as ETL has bright electroluminescence of up to 2714 cd/m^2^, while the Pe-LED device with B3PYMPM as ETL as higher peak luminous efficiency of 6.4 cd/A and peak luminous power efficiency of 5.7 lm/W. To our knowledge this is the first report on high brightness light emitting device based on FAPbBr_3_ nanocrystals in literature. Our studies indicate that the roll off in efficiency and the low performance in perovskite NC LED devices is attributed to the lattice changes due to charge injection and material degradation of the perovskite film. Hence, we believe better material engineering is essential to achieve higher efficiency in perovskite NC LED devices, our results clearly demonstrate that with the better perovskite material having lesser PL quenching at room temperature it should be possible to achieve much higher efficiency in Pe-LED devices.

## Methods

### Synthesis of (CH(NH_2_)_2_PbBr_3_ or FAPbBr_3_ NCs

0.1 mmol lead(II) bromide from Sigma Aldrich (36.7 mg) and 0.1 mmol formamidinium bromide from Dyesol (25.6 mg) is dissolved in 0.5 mL of DMF which we name it as precursor solution. In a beaker 5 mL toluene, 10 μL octylamine, 0.5 mL of oleic acid and 2 mL of butanol are added sequentially in that order and mixed on a magnetic stirrer at 800 rpm at room temperature. To this solution 150 μL of the precursor solution is added dropwise. The NCs are formed instantaneously, it is confirmed by color change – the transparent toluene solution turns bright yellowish green in color. Purification stage consists of 2 centrifuge steps. First centrifugation is performed at 7500 rpm for 5 minutes, this will remove all the unreactive materials in the supernatant and the supernatant is discarded and the bottom precipitate is used for further purification. 2 mL toluene is added for another centrifugation at 7500 rpm for 5 min. This time the supernatant is transferred to vial for further use in device fabrication.

### Perovskite film characterization

XRD data are collected on a Bruker Advance D8 X-ray diffractometer, using Cu-Kα radiation, at a scanning rate of 0.01° per step. X-ray photoelectron spectroscopy (XPS) and ultraviolet photoelectron spectroscopy (UPS) measurements are performed on the drop cast NC samples in UHV system. The XPS source is monochromatic Al Kα with photon energy at 1486.7 eV. The UPS source is from a helium discharge lamp (hν = 21.2 eV). The emitted photoelectrons are measured via an electron analyser (Omicron EA125). Small Angle X-ray Scattering (SAXS) experiments are performed using a SAXSess camera (Anton Paar, Graz, Austria) with a Cu anode X-ray source (PANalytical, PW3830) operating at 40 kV and 50 mA. Using a Goebel mirror and a collimation block the divergent polychromatic X-ray beam is collimated to a line-shaped beam of Cu Kα radiation (l = 0.154 nm). The perovskite nanocrystals are dispersed in toluene and the solution is held in a quartz capillary holder. The experiment is performed at room temperature and the scattering pattern is collected on an imaging plate. Raw data processing (Integration and background subtraction) is performed using SAXSQuant software from Anton-Paar. The SAXS data is evaluated using an indirect Fourier transform (GIFT software from Anton-Paar) to obtain the pair distance distribution function (PDDF) *p*(*r*), which can be used to determine the overall size and shape of the dispersed perovskite nanocrystals. TEM images are recorded on JEOL 2100F advanced field emission microscope operating at 200 kV accelerating voltage. The solution absorption is measured using Shimadzu spectrophotometer. The PL spectra are recorded with RF-5301PC fluorescence spectrometer (SHIMADZU). PLQE is measured using an integrated sphere and a fiber spectrometer (Ocean Optics USB 4000). The excitation source is 405 nm blue laser (Cobolt MLD^TM^). Temperature dependent steady state photoluminescence (PL) and time-resolved PL (TRPL) are performed with a Coherent Libra^TM^ femtosecond regenerative amplifier (1 kHz, ~50 fs) with frequency doubled 400 nm pulses at a pump fluence of 1.2 μJ cm^−2^ per pulse as the excitation source. The PL emission is collected in a conventional backscattering geometry and detected by a charge-coupled device array (Princeton Instruments, Pixis^TM^) coupled to a monochromator (Acton, Spectra Pro^TM^). The temporal evolution of the PL is resolved by an Optronis Optoscope^TM^ streak camera system.

### Device fabrication and characterization

ITO coated glass substrates with 10 ohm/square sheet resistance are purchased from Xiamen Weihua solar Co Ltd China. The substrates are sequentially washed with acetone, soap solution, ethanol and deionized water, followed by plasma treatment for 10 min at 100 W before use. The poly(3,4-ethylenedioxythiophene):poly(styrene-sulfonate) (PEDOT:PSS) Clevios Al 4043 Heraus GmbH solution is spin coated on the ITO film at 4000 rpm for 60 seconds, then it is annealed at 150 °C for 15 min. Then FAPbBr_3_ colloidal nanocrystals dispersed in toluene is spin-coated on the PEDOT:PSS film, followed by thermal annealing at 50 °C for 1 min. The substrates are then transferred to high vacuum thermal evaporator chamber, where 4,6-Bis(3,5-di(pyridin-3-yl)phenyl)-2-methylpyrimidine (B3PYMPM) or 2,2′,2″-(1,3,5-Benzinetriyl)-tris(1-phenyl-1-H-benzimidazole) TPBi (40 nm), Caesium carbonate (CsCO_3_) (5 nm) and Aluminium (Al) (80 nm) are sequentially thermally deposited. The active area of the devices fabricated in this work are 3 mm^2^. Devices are encapsulated before characterization.

The current–voltage–luminance characteristics is recorded via computer controlled source measure unit (Yokogawa GS610) and Konica Minolta LS-110 Luminance Meter. For measuring EQE of the LED device, Yokogawa GS610 source measure unit linked to a calibrated silicon photodiode is used to measure the current–voltage–luminance characteristics and the electroluminescence spectra of the LED device are recorded via a spectrophotometer PR-655 (Photo Research, Inc.). The lifetime test is performed via photodiode placed on top of the LED device and the photodiode is controlled via a source measure unit in a constant current mode.

## Additional Information

**How to cite this article**: Perumal, A. *et al.* High brightness formamidinium lead bromide perovskite nanocrystal light emitting devices. *Sci. Rep.* **6**, 36733; doi: 10.1038/srep36733 (2016).

**Publisher’s note**: Springer Nature remains neutral with regard to jurisdictional claims in published maps and institutional affiliations.

## Supplementary Material

Supplementary Information

## Figures and Tables

**Table 1:** Summarizes the FAPbBr_3_ NC film and solution PL decay lifetimes and amplitude values as a function of temperature.

Temp(K)	A_1_ (A1/A1 + A2 in %)	Τ_1_ (ns)	A_2_ (A2/A1 + A2 in %)	T_2_ (ns)	T-Average (Error)
**Thin film**					
**180**	22463 (47.5%)	1.1	24774.9 (52.5%)	8.21	4.8 (±0.05)
**210**	11886 (40.9%)	1.2	17169.8 (59.1%)	11.4	7.2 (±0.07)
**240**	7852.6 (38.5%)	0.97	12512.9 (61.5%)	11.9	7.7 (±0.07)
**270**	4264.7 (34.1%)	1.0	8228.3 (65.9%)	14.4	9.8 (±0.07)
**300**	2128.2 (32%)	0.99	4521.6 (68%)	14.6	10.2 (±0.11)
**330**	1042.7 (31.6%)	0.90	2252 (68.4%)	13.4	9.4 (±0.114)
**360**	591.3 (32.8%)	0.90	1208.1 (67.2%)	15.6	10.7 (±0.16)
**Solution (Room Temperature)**					
**Solution**	0.18 (20%)	5.45	0.70 (80%)	69.29	56

**Table 2:** Summarizes the device performance values for QD LED device and the Pe-LED device.

Voltage (V)	Current density (mA/cm^2^)	Luminance (cd/m^2^)	Cd/A	lm/W
QD LED device				
6.6	0.54	92	17.1	8.1
7.6	6.46	1357	21.0	8.1
8.8	49.12	10550	21.4	7.6
Perovskite LED with FAPbBr_3_ NCs				
2.8	5.8	258	4.4	4.9
3.8	98.3	2714	2.7	2.2
