# Supplementary material for: High brightness formamidinium lead bromide perovskite nanocrystal light emitting devices
Source: Sci Rep. 2016 Nov 9;6:36733. doi: 10.1038/srep36733 (PMC5101487; doi:10.1038/srep36733)
Supplement: Supplementary Information [file srep36733-s1.pdf]

Supplementary Info for “High brightness formamidinium lead bromide perovskite nanocrystal light emitting devices”

*Ajay Perumal[1] *, Sushant Shendre[1], Mingjie Li[2], Yong Kang Eugene Tay[2], Vijay Kumar Sharma[1,2,4], Shi Chen[2], Zhanhua Wei[2], Qing Liu[3], Yuan Gao[1], Pio John S Buenconsejo[3], Swee Tiam Tan[1], Chee Lip Gan[3], Qihua Xiong[2], Tze Chien Sum[2] and Hilmi Volkan Demir[1,2,4]**

[1]LUMINOUS! Center of Excellence for Semiconductor Lighting and Displays, School of Electrical and Electronic Engineering, School of Mathematical and Physical Sciences, Nanyang Technological University, Singapore 639798

[2]Division of Physics and Applied Physics, School of Physical and Mathematical Sciences, Nanyang Technological University, Singapore 637371

[3]School of Materials Science and Engineering, Nanyang Technological University, Singapore 639798, Singapore

[4]UNAM-Institute of Materials Science and Nanotechnology, Department of Electrical and Electronics Engineering, Department of Physics, Bilkent University, Ankara, Turkey 06800

KEYWORDS: Perovskite LED; Formamidinium lead bromide; $FAPbBr_3$ nanocrystals; Organic-inorganic halide perovskites

X-Ray Diffraction Analysis:

Table ST1:

Cubic phase indexing

Space group Pm-3m

Cry size Lorentzian (nm) 18.03(30)

Pawley method

Phase name FAPbBr3_hkl_Phase

R-Bragg-15.212

Cell Mass 627.003

Cell Volume (Å^3) 216.661(21)

Lattice parameters: a (Å) 6.00611(20)

h	k	l	d-spacing()	2Theta()	Intensity
0	0	1	6.00611	14.73723	104.24(55)
0	1	1	4.24696	20.89986	36.53(59)
1	1	1	3.46763	25.66947	11.79(71)
0	0	2	3.00306	29.72553	298.8(18)
0	2	1	2.68601	33.33067	142.1(16)
2	1	1	2.45198	36.61936	14.1(13)
0	2	2	2.12348	42.53866	106.7(20)
0	0	3	2.00204	45.25732	
0	3	1	1.8993	47.85365	30.2(19)
3	1	1	1.81091	50.34753	12.9(20)
2	2	2	1.73382	52.75446	42.7(24)
0	3	2	1.6658	55.08681	42.9(26)
3	2	1	1.6052	57.35466	18.0(28)
0	0	4	1.50153	61.72899	37.4(35)
0	4	1	1.4567	63.84856	
0	3	3	1.41565	65.93028	
3	3	1	1.3779	67.97874	0.0(40)
0	4	2	1.34301	69.99798	35.1(45)
4	2	1	1.31064	71.99163	56.0(48)

3	3	2	1.28051	73.96297	29.5(48)
4	2	2	1.22599	77.85046	10.9(59)
0	0	5	1.20122	79.77195	

Tetragonal phase Indexing

Space group		P4mm	
Cell	P4mm	(Å^3)	216.6(10)
**a**	**P4mm**	**6.006 Å (10)**	
**c**	**P4mm**	**6.007 Å (20)**	

h	k	l	d-spacing ()	2-theta ()
0	0	1	6.00673	14.73571
0	1	0	6.00559	14.73851
0	1	1	4.247	20.89969
1	1	0	4.24659	20.90169
1	1	1	3.46755	25.67008
0	0	2	3.00336	29.72242
0	2	0	3.0028	29.72816
0	1	2	2.68619	33.32846
0	2	1	2.68588	33.33235
2	1	0	2.68578	33.33364
1	1	2	2.45208	36.61786
2	1	1	2.45185	36.62145
0	2	2	2.1235	42.53831
2	2	0	2.1233	42.54252
0	0	3	2.00224	45.25243
2	1	2	2.00203	45.25744
2	2	1	2.00191	45.26045
0	3	0	2.00186	45.26146
0	1	3	1.89946	47.8494

0	3	1	1.89917	47.85709
3	1	0	1.89913	47.85805
1	1	3	1.81103	50.34386
3	1	1	1.81079	50.35127
2	2	2	1.73377	52.7558
0	2	3	1.66587	55.08417
0	3	2	1.66575	55.08851
3	2	0	1.66565	55.09199
2	1	3	1.60526	57.35247
3	1	2	1.60515	57.3567
3	2	1	1.60508	57.35924
0	0	4	1.50168	61.72198
0	4	0	1.5014	61.73492
0	1	4	1.45683	63.84204
2	2	3	1.45672	63.8476
3	2	2	1.45663	63.85156
0	4	1	1.45659	63.85394
4	1	0	1.45657	63.85474
1	1	4	1.41577	65.92423
0	3	3	1.41567	65.9297
4	1	1	1.41555	65.93594
3	3	0	1.41553	65.93671
3	1	3	1.3779	67.97851
3	3	1	1.37779	67.98466
0	2	4	1.34309	69.99279
0	4	2	1.34294	70.00189
4	2	0	1.34289	70.00493
2	1	4	1.31072	71.98685
4	1	2	1.31057	71.99584
4	2	1	1.31054	71.99809
3	2	3	1.2805	73.96377

3	3	2	1.28044	73.96748
2	2	4	1.22604	77.84681
4	2	2	1.22592	77.85555
0	0	5	1.20135	79.76214
0	3	4	1.20126	79.76865
0	4	3	1.2012	79.77372
0	5	0	1.20112	79.78024
4	3	0	1.20112	79.78024
0	1	5	1.17801	81.67245
3	1	4	1.17793	81.67892
4	1	3	1.17787	81.68397
0	5	1	1.1778	81.68973
4	3	1	1.1778	81.68973
5	1	0	1.17779	81.69045
1	1	5	1.15598	83.57347
3	3	3	1.15585	83.58495
5	1	1	1.15578	83.59068
0	2	5	1.11539	87.35641
3	2	4	1.11533	87.36283
4	2	3	1.11528	87.36782
0	5	2	1.11524	87.37139
4	3	2	1.11524	87.37139
5	2	0	1.11521	87.37424
2	1	5	1.09664	89.24253
5	1	2	1.09649	89.2575
5	2	1	1.09647	89.25964

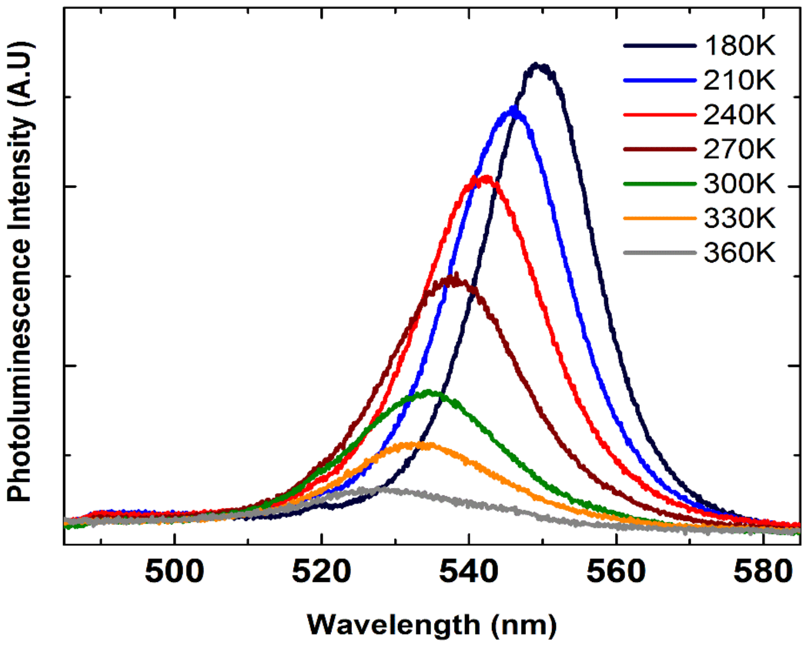

***Figure S1**: The absolute PL emission spectra under a pump fluence of 1.2 μJ cm^{-2} at temperatures ranging from 180-360K.*

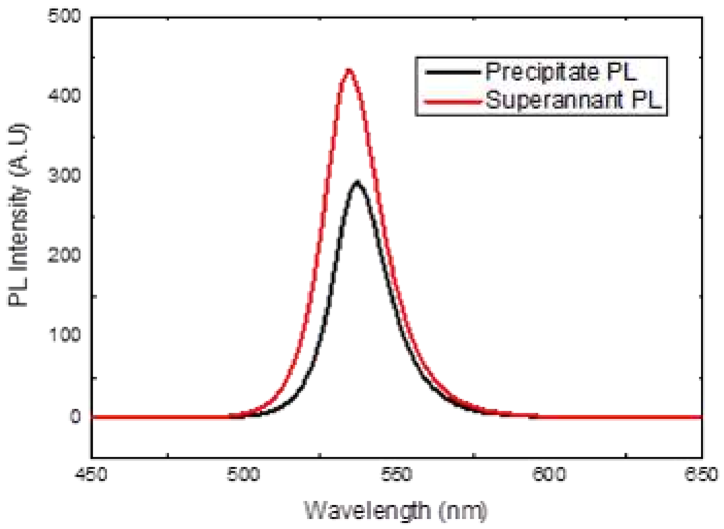

***Figure S2**:* PL emission measurements of supernatant (having nanocrystals of smaller size) and the precipitate (having nanocrystals of much larger size compared to supernatant) their emission peaks are not much different.

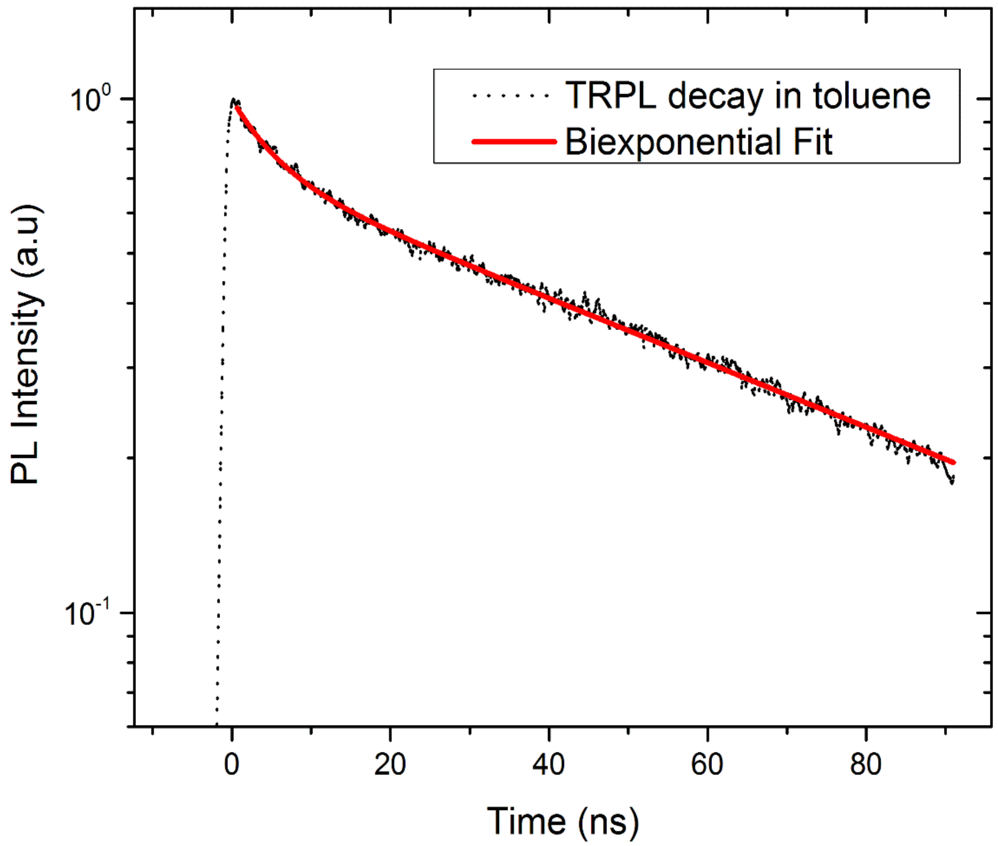

Figure S3*: The solution TRPL of FAPbBr3 in toluene recorded at room temperature along with the exponential fit.*

Ionization potential for MAPbBr3 - 6.25eV   CB – 3.9eV
Ionization potential for FAPbBr3 - 6.55eV   CB – 4.2eV

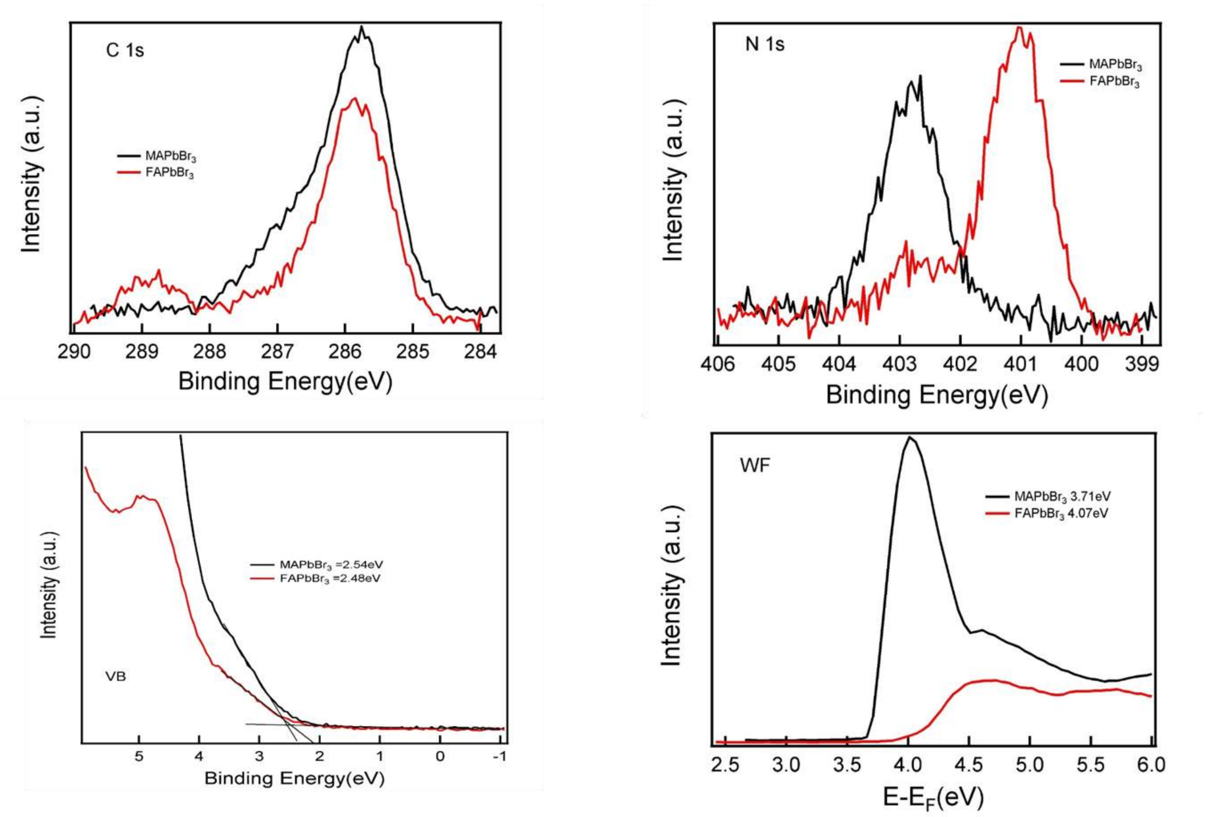

Figure S4: *XPS and UPS spectra on FAPbBr3 NCs drop cast on a substrate.*

The Carbon 1s peak in The MAPbBr3 and FAPbBr3 samples are similar, the main peak at around 285.8eV in both spectra is originated from the carbon atoms in MA and FA cations. The N 1s peak show a 2eV difference between MAPbBr3 (~403eV) and FAPbBr3 (~401eV) samples. The higher N 1s binding energy in MAPbBr3 sample is due to the stronger C-N bonding in MA cation. The valence band relative to the Fermi level E_F and the Fermi level E_F relative to vacuum are also shown. We deduce the ionization potential to be 6.55eV and the CB is at 4.2eV for $FAPbBr_3$ NC film.

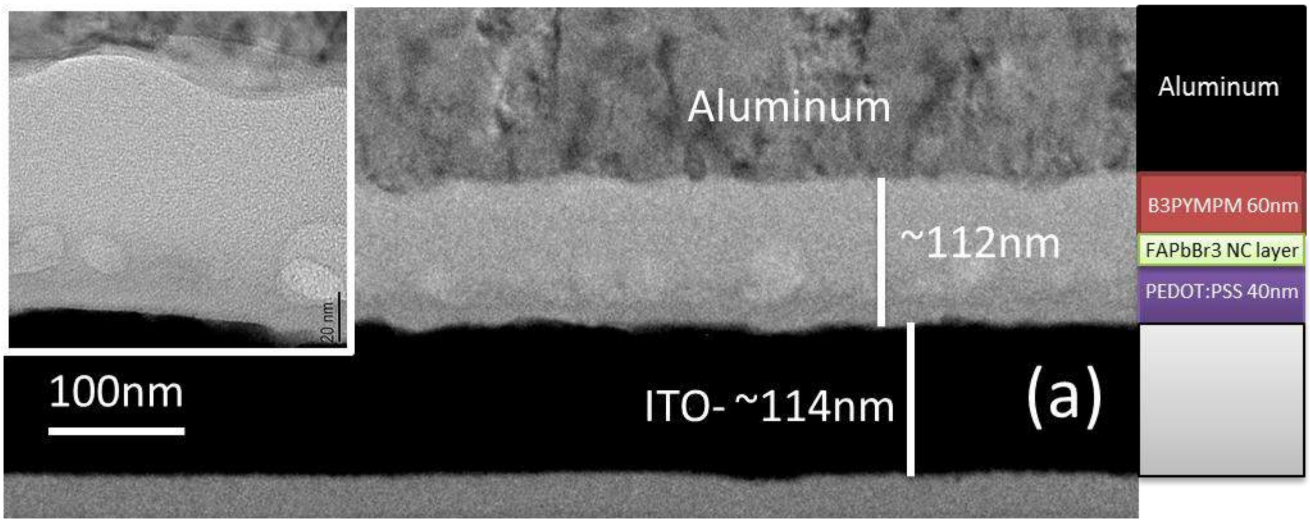

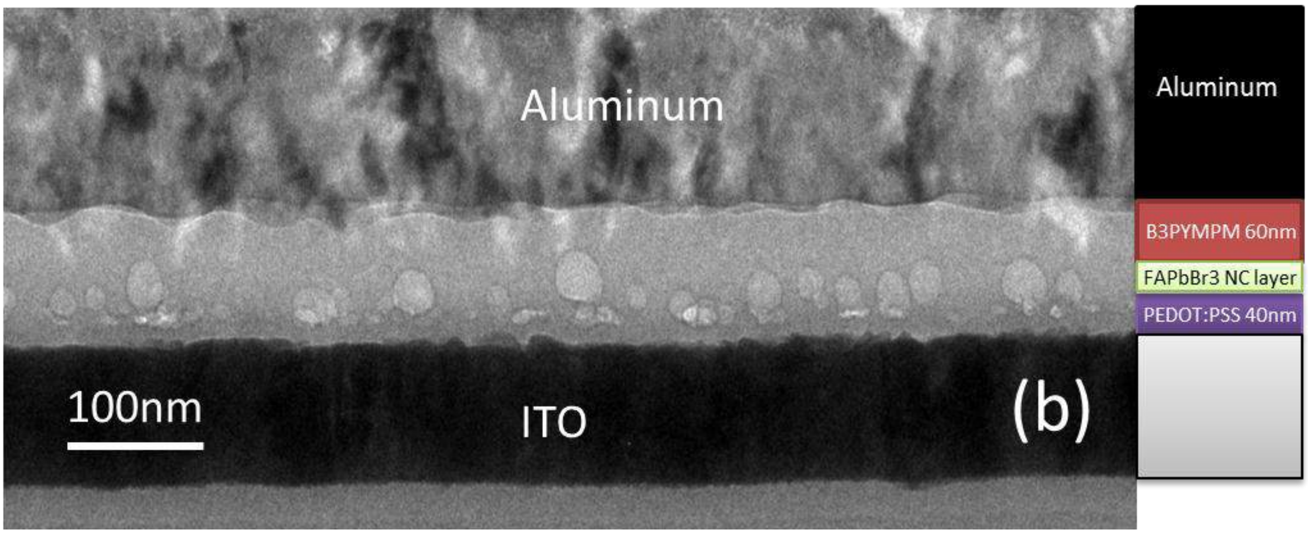

***Figure S5**: Cross sectional Bright field TEM image of the full Pe-LED device with TEM specimen is prepared via FIB technique. In general the organic layers and the perovskite are dominated by carbon, nitrogen. Hence we could not see any distinguishable contrast for different active layers. The PEDOT:PSS, $FAPbBr_3$ and the B3PYMPM layer appear to be of same contrast with total thickness to be 112 nm.*

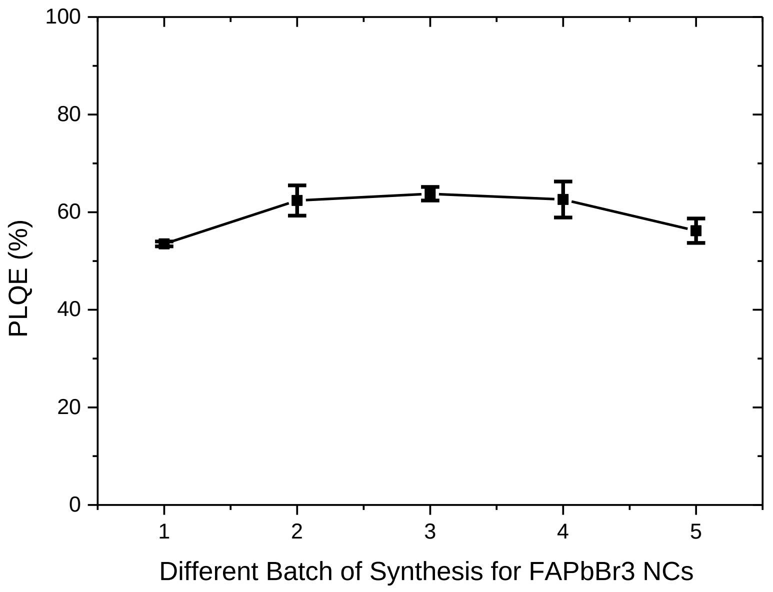

Figure S6: *PLQE for 5 different batch synthesis of $FAPbBr_3$ NCs.*

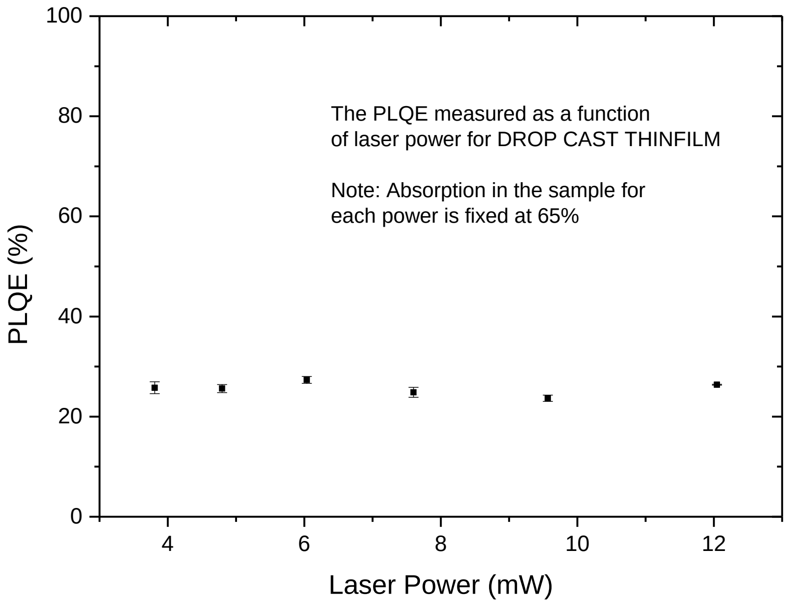

Figure S7: *PLQE variation as a function of laser power.*

Small angle X-Ray scattering (SAXS) technique Description:

Small angle X-ray scattering (SAXS) is a common method to determine the shape and size distribution of particles in colloidal solution, and this technique is unique due to its sub nanometer resolution (1-100 nm) and also it probes large sample sizes (averaging over large number of nanocrystals) [1, 2]. In a liquid sample probe, the measured scattering signal originates from the contrast between the nanocrystals and the solvent. When a particle with the electron density of ρ_1 is embedded into a matrix of electron density ρ_2 , then the scattered intensity for poly-disperse sample with N particles is [2]

$$\Delta I(q) = I_0 \sum_{i=1}^{N} (\Delta\rho)_i^2 \; V_i^2 \; P_i(q) S_i(q)$$

Where V is the particle volume, $\Delta\rho = \rho_1 - \rho_2$ and P(q) is the form factor of the particle. For dilute solutions as is the case with our liquid sample suspension of NCs, the particle interactions can be assumed to be negligible S(q)=0 [2] then every particle in the liquid suspension produces a form factor that is characteristic to its structure [3]. The oscillating part of the form factor is represented in "real space" via Fourier transformation resulting in a pair distance distribution function p(r) which is related with the experimental form factor P(q) via

$$P(q) = \int_0^\infty p(r) \frac{\sin qr}{qr} dr$$

The pair distance distribution function is a histogram of size of the particles in liquid suspension in our sample.

1. Bandyopadhyay, J.; Sinha Ray, S. *Polymer* **2010,** 51, (6), 1437-1449.
2. Fedeyko, J. M.; Vlachos, D. G.; Lobo, R. F. *Langmuir* **2005,** 21, (11), 5197-5206.
3. Hanrath, T. *Journal of Vacuum Science & Technology A: Vacuum, Surfaces, and Films* **2012,** 30, (3), 030802.
